# New Insights Into Implications of CTRP3 in Obesity, Metabolic Dysfunction, and Cardiovascular Diseases: Potential of Therapeutic Interventions

**DOI:** 10.3389/fphys.2020.570270

**Published:** 2020-12-03

**Authors:** Bei Guo, Tongtian Zhuang, Feng Xu, Xiao Lin, Fuxingzi Li, Su-Kang Shan, Feng Wu, Jia-Yu Zhong, Yi Wang, Ming-Hui Zheng, Qiu-Shuang Xu, Ullah Muhammad Hasnain Ehsan, Ling-Qing Yuan

**Affiliations:** ^1^National Clinical Research Center for Metabolic Diseases, Hunan Provincial Key Laboratory of Metabolic Bone Diseases, and Department of Endocrinology and Metabolism, The Second Xiangya Hospital, Central South University, Changsha, China; ^2^Department of Dermatology, Xijing Hospital, Fourth Military Medical University, Xi’an, China; ^3^Department of Radiology, The Second Xiangya Hospital, Central South University, Changsha, China; ^4^Department of Pathology, The Second Xiangya Hospital, Central South University, Changsha, China

**Keywords:** adipocytokines, CTRP3, insulin resistance, therapeutic targets, type 2 diabetes mellitus, obesity, metabolic syndrome, cardiovascular disorders

## Abstract

Adipose tissue, as the largest endocrine organ, secretes many biologically active molecules circulating in the bloodstream, collectively termed adipocytokines, which not only regulate the metabolism but also play a role in pathophysiological processes. C1q tumor necrosis factor (TNF)-related protein 3 (CTRP3) is a member of C1q tumor necrosis factor-related proteins (CTRPs), which is a paralog of adiponectin. CTRP3 has a wide range of effects on glucose/lipid metabolism, inflammation, and contributes to cardiovascular protection. In this review, we comprehensively discussed the latest research on CTRP3 in obesity, diabetes, metabolic syndrome, and cardiovascular diseases.

## Introduction

Adipose tissue (AT) is widely known as an endocrine organ and a sensor/regulator of energy homeostasis (Rosen and Spiegelman, 2006; Scherer, 2006). Recently, it has been widely accepted that changes in the biological function of AT, rather than just changes in AT mass, play an important role in the physiological and pathological processes. AT secretes unique adipokines through endocrine and paracrine pathways to regulate systemic or local metabolism (Akoumianakis and Antoniades, 2017). Adipocytokines play diverse and important roles in the occurrence and progression of insulin resistance in obesity, metabolic syndrome (MS), type 2 diabetes mellitus (T2DM), and cardiovascular disorders (CVD) (Akoumianakis and Antoniades, 2017).

CTRPs are a family of adipocytokines. Due to the same modular organization, CTRPs members and adiponectin are classified into the C1q/TNF superfamily (Shapiro and Scherer, 1998; Kishore et al., 2004). CTRP3, as a novel member of CTRPs, was discovered in 2001. CTRP3 has multiple effects on the metabolism (Peterson et al., 2010, 2013; Zhou et al., 2014), inflammation (Weigert et al., 2005), proliferation (Yi et al., 2012), apoptosis (Song et al., 2020), vascular calcification (Zhou et al., 2014), fibrosis (Wu et al., 2015), ischemic injury (Zhang Z. et al., 2019), etc. (Table 1) (Akiyama et al., 2009; Kim et al., 2012; Hou et al., 2015). In this review, we summarize the role of CTRP3 in obesity, MS, T2DM, and CVD.

**TABLE 1:** Effects of CTRP3 on signals or critical factors in metabolism and the cardiovascular system.

Signals or critical factors	References	Effects
↑PI3K/AKT	Shepherd et al., 1993; Akiyama et al., 2007; Peterson et al., 2010; Yi et al., 2012	Promotes angiogenesis, reduces insulin resistance, inhibits gluconeogenesis in liver, and suppresses glucose uptake in cultured adipocyte and muscle cells
↑ERK1/2	Akiyama et al., 2007; Maeda and Wakisaka, 2010; Zhang Z. et al., 2019	Promotes angiogenesis, myocardiocytes proliferation and migration
↑AMPK	Zheng et al., 2011; Liu et al., 2012; Peterson et al., 2013; Ma et al., 2017; Zhang B. et al., 2019	Regulates lipid metabolism, vasorelaxation, increases mitochondrial biogenesis, attenuates cardiomyopathy
↑Adiponectin, Leptin	Pajvani et al., 2003; Li et al., 2014a	Reduces triacylglycerol synthesis
↓C/EBP α,PPAR γ,FABP4	Nishimoto et al., 2017	Inhibits adipogenesis
↓TGF β/smad3	Yi et al., 2012; Lin et al., 2014	Attenuates vascular fibrosis
↓IL-6, TNFα	Alberti and Zimmet, 1998; Cherrington, 1999; Kopp et al., 2010; Peterson et al., 2013; Wolf et al., 2016	Reduces insulin resistance and anti-inflammatory effect
↓LPS/TLR4	Kopp et al., 2010	Inhibit inflammatory response
↓p38 MAPK	Zhang B. et al., 2019	Inhibits ER stress
↑PKA	Li et al., 2014b, 2015	Increase CTRP3 expression
↑PKA -TAK1-c-JNK	Ma et al., 2019	Exacerbates cardiac hypertrophy
↑mTOR	Rehorst et al., 2019	Enhances axonal outgrowth
↓JAK2/STAT3	Hu et al., 2019	Inhibits high glucose-induced proliferation and extracellular matrix production in human glomerular mesangial cells

*CTRP3, C1q tumor necrosis factor-related protein 3; ER, endoplasmic reticulum; PI3K, phosphatidylinositol-4,5-bisphosphate 3-kinase; ERK1/2, extracellular signal-regulated kinase 1/2; AMPK, adenosine 5′-monophosphate-activated protein kinase; C/EBPα, CCAAT/enhancer binding protein alpha; PPARγ, peroxisome proliferator-activated receptor gamma; FABP4, fatty acid binding protein 4; TGF-β, transforming growth factor β; IL-6, interleukin-6; TNF-α, tumor necrosis factor α; LPS, lipopolysaccharide; TLR4, Toll-like receptor 4; MAPK, mitogen-activated protein kinase; PKA, protein kinase A; TAK1, transforming growth factor β-activated kinase 1; JNK, c-Jun N-terminal kinase; mTOR, mammalian target of rapamycin; JAK, just another kinase or janus kinase; STAT3, signal transducers and activators of transcription 3.*

### C1Q/TNF-RELATED PROTEIN-3 (CTRP3)

**FIGURE 1:**
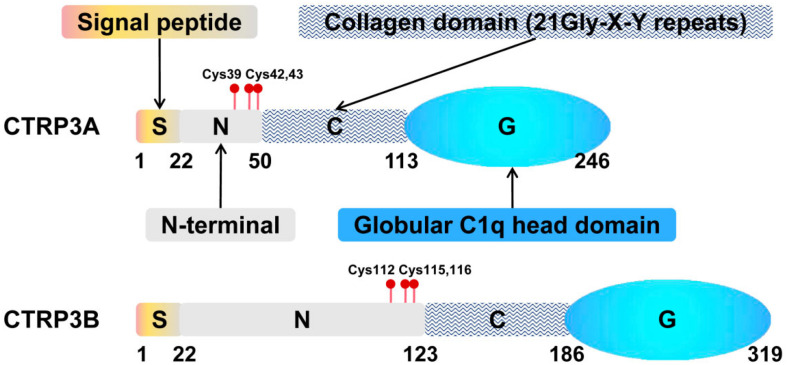
Structural organization of CTRP3. Domain structure of a CTRPA and CTRPB monomeric protein. S, N-terminal signal peptide; N, N-terminal; C, collagen domain containing collagen triplets (Gly-X-Y); G, C-terminal globular complement factor C1q domain.

CTRP3 was named as CORS26 (collagenous repeat-containing sequence of 26-kDa protein) when it was first discovered by Maeda T. et al. (Maeda et al., 2001). Since the expression of CTRP3 in developing cartilage, cartducin (Akiyama et al., 2006; Maeda et al., 2006) and cartonectin (Schaffler et al., 2007; Wolfing et al., 2008) are used as alternative names for CTRP3. Subsequently, Wong and colleagues identified CTRP3 as a novel member of highly conserved Acrp30/adiponectin paralogs which are designated as CTRPs (Wong et al., 2004). The CTRPs is a secreted molecule consisting of 4 different domains: an N-terminal signal peptide, a short variable domain, a collagen-like domain, and a C-terminal C1q-like globular domain (Kishore et al., 2004; Wong et al., 2004). Figure 1 shows the structure of CTRP3 (Shapiro and Scherer, 1998; Maeda et al., 2001). Two splice variants of CTRP3, CTRP3A, and CTRP3B, have been identified, which differ in length and glycosylation (Figure 1; Peterson et al., 2010). Moreover, the N-terminal Cys residue of CTRP3A and CTRP3B are different: CTRP3A (Cys-39, -42, and -43) and CTRP3B (Cys-112, -115, and -116) (Figure 1). The CTRP3A and CTRP3B were found in both human serum and mouse AT, while only CTRP3A could be detected in mouse serum (Wong et al., 2008; Peterson et al., 2010). CTRP3B degrades rapidly unless it forms a higher-order molecular polymer with CTRP3A (Peterson et al., 2010), which increases the difficulty of CTRP3B detection. However, this low-molecular-weight polymer only occurs between its own two splice variants (Peterson et al., 2010), while most CTRPs form high-order oligomers with adiponectin or at least one other CTRP when co-expressed (Wong et al., 2004, 2008).

Recently, it has been reported that the possible receptors of CTRP3 are lysosomal-associated membrane protein 1 (LAMP-1) and lysosome membrane protein 2 (LIMP II), both of which are widely expressed in many different tissues (Li et al., 2016). However, the definite mechanism of these two receptors in different tissues remains unclear. Another study shows that CTRP3 specifically inhibits the binding of lipopolysaccharide (LPS) to its receptor, toll-like receptor 4 (TLR4)/MD-2 (Kopp et al., 2010). LPS is a potent endotoxin, and the TLR4/MD-2 fusion molecule is an LPS trap, which has been proven to bind mutually (Brandl et al., 2005). However, CTRP3 doesn’t directly bind to LPS or TLR4, so their interaction mechanism is still unknown.

Previously, the putative transcription binding sites of the promoter region of the CTRP3 gene were analyzed, including the locus of the following transcription factors: activator protein 1 (AP-1), POU domain, class 1, peroxisome proliferator-activated receptor (PPAR), transcription factor 1 (Pit-1a), specificity protein 1 (SP-1), Fos proto-oncogene (c-FOS), c-Jun N-terminal kinase (c-Jun), similar to myelocytomatosis viral oncogene (c-Myc), cAMP response element-binding protein (CREB), etc. However, only a few transcription factors regulate CTRP3 expression (Schaffler et al., 2003a, b, 2004). SP-1, Pit-1, and PPARγ can specifically bind to the CTRP3 promoter and inhibit its transcriptional activation in mature adipocytes (Schaffler et al., 2004, 2007). Classic AP-1 is a heterodimer composed of c-Fos and c-Jun. The c-Fos and c-Jun bind to two cooperative motifs -HOB1 and -HOB2, respectively in the AP-1 region (Bannister et al., 1994). A study shows that c-Jun can directly bind to the AP-1 binding site of the CTRP3 promoter and increase its transcriptional activation in chondrocytes (Kim et al., 2012).

## CTRP3 Expression *in vitro* and *in vivo*

It is reported that many cell lines express CTRP3, such as mouse chondrogenic progenitor (N1511) (Maeda et al., 2006), mouse preadipocytes (3T3-L1a) (Schaffler et al., 2007; Schmid et al., 2012; Li et al., 2015), mouse osteoblast-like non-cancer cell line (MC3T3-E1) (Akiyama et al., 2006), mouse osteosarcoma cell line (LM8, NOHS) (Akiyama et al., 2009), TM3 mouse Leydig cells (Otani et al., 2012), and human mesangial cells (HMCs) (Zhang et al., 2016). Even so, CTPR3 is mainly secreted by AT, and considered as an adipokine (Schaffler et al., 2003a, 2007; Wong et al., 2008). The CTRP3 can also be detected in many tissues and organs, including heart, liver, kidney, lung, bone and cartilage, blood vessels (Maeda et al., 2001, 2006; Schaffler et al., 2003a; Wong et al., 2004, 2008; Akiyama et al., 2006; Peterson et al., 2010; Otani et al., 2012; Schmid et al., 2012; Zhou et al., 2014). These studies reveal that CTRP3 may play important roles in metabolism, inflammation, bone growth and development, and certain types of cancer. Next, we will highlight the impacts and therapeutic potentials of CTRP3 on obesity, MS, T2DM, and CVD.

## CTRP3 in Obesity and Metabolic Syndrome

Excessive fat accumulation can lead to obesity, which is a pandemic social problem worldwide. Obesity can change the endocrine function of AT (Weisberg et al., 2003) and cause chronic low-grade inflammation, which initiates and promotes the development of metabolic diseases (Donath and Shoelson, 2011; Moore and Tabas, 2011; Ouchi et al., 2011). A common and major risk factor for MS is chronic low-grade inflammation, which is characterized by an increase in circulating pro-inflammatory cytokines such as IL-6, TNF, and C-reactive protein (CRP). Furthermore, MS increases the risk of T2DM, CVD, and mortality (Alberti and Zimmet, 1998; Kaur, 2014). AT, as an active endocrine organ, secretes various adipocytokines (Ouchi et al., 2000, 2003, 2011) which are roughly divided into anti-inflammatory adipocytokines and pro-inflammatory adipocytokines according to their effects on inflammation. CTRP3 is one of the anti-inflammatory adipocytokines and inversely correlates with the pro-inflammatory cytokines (IL-6, TNF, and CRP) (Choi et al., 2012, 2014; Peterson et al., 2013; Tan et al., 2013; Yoo et al., 2013; Ban et al., 2014; Deng et al., 2015; Qu et al., 2015; Petersen et al., 2016). The following section in obesity and MS mainly focuses on the role of CTRP3 in obesity and MS.

*In vivo* experiments have shown that diet-induced obesity (DIO) leads to decreased CTRP3 levels and increased TNF and IL-6 levels (Peterson et al., 2010). Consistently, CTRP3-knockout in DIO mice markedly increases IL-6 and TNF levels (Wolf et al., 2016). On another note, thromboxane A2 (TXA2) is essential for regulating platelet aggregation, but excessive TXA2 levels are related to the occurrence and development of insulin resistance (IR) and arteriosclerosis (Graziani et al., 2011). It is worth noting that the level of TXA2 increased in DIO mice and obese subjects, whereas the loss of thromboxane synthase (a key enzyme for TXA2 synthesis) significantly improved insulin sensitivity with decreased IL-6 levels in circulation, and elevated CTRP3 and other CTRP levels (CTRP9, CTRP12) (Lei et al., 2015). In addition, LPS inhibits CTRP3 expression *in vitro* (Schmid et al., 2012). CTRP-3 exhibits potent anti-inflammatory effects on LPS-induced inflammatory reaction in mature 3T3-L1 adipocytes via reducing the release of pro-inflammatory monocyte chemoattractant protein-1 (MCP-1) (Kopp et al., 2010) which attract monocytes, causing macrophage accumulation in AT of obese subjects (Weisberg et al., 2003). In addition, *Ex vivo* data revealed that CTRP-3 also has anti-inflammatory effects on LPS-induced primary monocytes isolated from T2DM patients (*n* = 30) and healthy subjects (*n* = 20), including decreasing the levels of MCP-1, CC-chemokine ligand (CCL)3/macrophage inflammatory protein (MIP)-1, CCL4, and macrophage migration inhibitory factor (MIF) (Kopp et al., 2010). Besides, CTRP-3 reduces lauric acid (a saturated fatty acid)-induced inflammatory activation in primary human monocytes possibly involving p38 MAPK and ERK1/2 pathways (Kopp et al., 2010). *In vivo*, intraperitoneal injection of CTRP3 significantly reduces the LPS-induced inflammatory response (Ouchi et al., 2011), and further studies are needed to explore clinically administered doses of CTRP3. In the human study, CTRP3 significantly inhibits the production of IL-6 induced by LPS in monocytes derived from healthy controls, but CTRP3 had no such effect on IL-6 in monocytes isolated from diabetic patients (Alberti and Zimmet, 1998; Cherrington, 1999). Additionally, CTRP3 also influences some specific inflammatory disorders, such as immunoglobulin A nephropathy (Zhang et al., 2016) and inflammatory bowel disease (Zhang et al., 2016; Mohamadinarab et al., 2020). Overall, CTRP-3 is a promising target for the treatment of chronic low-grade inflammation-related diseases.

In addition to its role in inflammation, CTRP3 affects adipocytes differentiation and adipogenesis. Knocking down CTRP3 in adipocytes reduces the size of lipid droplets and triglyceride (TG) in cells, indicating that CTRP-3 deficiency appears to induce the dedifferentiation of mature adipocytes (Kopp et al., 2010). Consistently, CTRP3 is not detected in 3T3-L1 preadipocytes and the first 2-day of adipocytes differentiation and is detectable until the 4th day (Schaffler et al., 2007). Paradoxically, another study reported that CTRP3 levels were significantly reduced during the adipocyte differentiation of 3T3-L1 cells (Nishimoto et al., 2017). The treatment with recombinant CTRP3 (rCTRP3) significantly reduce lipid and the expression of adipogenesis genes, such as PPARγ, fatty acid-binding protein 4 (FABP4), and adiponectin in 3T3-L1 cells (Nishimoto et al., 2017). Unfortunately, there are few studies on the effect of CTRP3 on adipogenic differentiation, and corresponding research is needed.

Some studies investigate the association between obesity and circulating CTRP3 levels in patients. The level of CTRP3 in the tissue is about one-tenth of the level of circulating adiponectin, and the level of CTRP3 in obese patients is further reduced (Peterson et al., 2010). Indeed, a cross-sectional study reiterated this result (Wolf et al., 2015). In obese children, the level of CTRP3 decreased significantly, and its level was negatively correlated with IR and pancreatic β-cell function indicators (Zhang and He, 2019). Choi et al. examine the changes in the circulating CTRP3 before and after 3 months of exercise in 76 obese Korean females (Choi et al., 2013). The results show that exercise leads to a 15% reduction in CTRP3 levels and 9% weight loss. However, the relationship among exercise, diet control, weight change is complex and challenging to define. Moreover, the circulating CTRP3 inversely correlates with non-alcoholic fatty liver disease (Zhang et al., 2018; Zhou et al., 2018).

Based on the current data, the results of most studies are consistent. CTRP3 is reduced in the blood of obese patients, and it decreases the production of inflammatory factors, inhibits the activation of inflammatory signals, and reduces lipid formation. Overall, these findings suggest that CTRP3 is a potential target in the treatment of patients with MS or obesity.

## CTRP3 in Type 2 Diabetes Mellitus

Over the past 50 years, approximately 693 million people worldwide suffer from diabetes mellitus (DM), which has caused huge economic losses (Cho et al., 2018). Researchers have been seeking to treat diabetes. It is reported that adipocytokine CTRP3 has a positive effect on glucose metabolism. An *in vivo* experiment indicates that an acute administration of rCTRP3 decreases serum glucose for up to 8-h, but not influences insulin levels in rodents. Intriguingly, the chronic transgenic overexpression of CTRP3 doesn’t influence glucose levels, demonstrating the existence of a potential compensatory mechanism (Peterson et al., 2010). Transgene overexpression or daily CTRP3 injection is sufficient to reduce HFD-induced liver IR and liver steatosis (Peterson et al., 2013).

Although CTRP3 plays a beneficial role in the regulation of IR and serum glucose, the discrepancy for CTRP3 levels still exists in different studies. Several cross-sectional human studies show that the correlation between CTRP3 and some metabolic indicators appears to evolve dynamically from the period of pre-diabetes to the clinical diagnosis of diabetes. In nondiabetic Korean adults, Choi et al. (Choi et al., 2013) demonstrate that CTRP3 levels are independently associated with adiponectin, TG, low-density lipoprotein (LDL), and retinol-binding protein 4. In pre-diabetic adults, CTRP3 levels are positively correlated with total cholesterol, CRP, and fasting blood glucose (FBG). In newly diagnosed T2DM, serum CTRP3 was significantly reduced after 2 h of 75 g OGTT (Ban et al., 2014). However, in adults with T2DM, CTRP3 levels are inversely correlated with IL-6, HbA1c, and HOMA-IR (Qu et al., 2015; Yan et al., 2017). When we focus on the changes of CTRP3 levels, we found that it was significantly increased in subjects with pre-diabetic subjects (Choi et al., 2012), and decreased significantly in newly diagnosed T2DM (Ban et al., 2014) or T2DM (Deng et al., 2015; Qu et al., 2015; Moradi et al., 2019). Consistently, in the T2DM rat model, the expression of CTRP3 gradually decreases from the IR stage to the clinical stage of diabetes in visceral AT (Li et al., 2014b). Based on the above evidence, we speculate that in the pre-diabetes stage, before CTRP3 is insufficient to compensate for the severe metabolic disorders of T2DM and/or obesity, the level of CTRP3 may increase through a compensation mechanism. Then it declines like other adipocytokines. However, why the changes of CTRP3 levels after anti-diabetic drugs or insulin treatment in diabetic patients are inconsistent? Briefly, several studies illustrate that CTRP3 levels are increased with the treatment of metformin (Flehmig et al., 2014) or GLP-1 receptor agonist (Li et al., 2014b, 2015), indicating that at least antidiabetic drugs affect CTRP3 levels. Whereas, a 6-month insulin mixture therapy significantly decreases the plasma CTRP3 in obese patients with T2DM, compared with the levels prior to insulin treatment (Komosinska-Vassev et al., 2019). Is there an undiscovered competitive mechanism between CTRP3 and insulin? Notably, a divergent point is the influence of gender on CTRP3 levels. Most studies have shown that women have higher circulating CTRP3 levels than men (Choi et al., 2013; Yoo et al., 2013; Deng et al., 2015; Wolf et al., 2015), with one exception (Qu et al., 2015). Wager and colleagues found that obese men had elevated CTRP3 levels, while obese women had lower CTRP3 levels (Wagner et al., 2016). The gender-dependent phenotype changes in CTRP3 levels may explain the contrary data, as the proportion of men and women in each study is different. In any case, more clinical researches are needed. Interestingly, unlike most cytokines, circulating CTRP3 levels increase during fasting (Peterson et al., 2010), which suggests that CTRP3 may be activated by the glucagon pathway or inhibited by the insulin pathway. In summary, CTRP3 regulates blood glucose homeostasis and IR. Therefore, we summarize some related mechanisms as follows.

1. CTRP3 regulates hepatic glucose output through activating AKT and ERK1/2 pathways in the liver, thereby inhibiting the expression of gluconeogenic enzymes G6Pase and PEPCK *in vitro* and *in vivo* (Peterson et al., 2010). This notion is consistent with our known knowledge: AKT activation effectively suppresses hepatic gluconeogenesis in the liver (Liao et al., 1998; Miyake et al., 2002; Ono et al., 2003) by increasing Foxo1 phosphorylation, thus p-Foxo1 is excluded from the nucleus to keep gluconeogenic genes from the transcription (Gross et al., 2009); ERK1/2 also regulates transcription factors, such as Foxo1 (Asada et al., 2007), but the effect of ERK1/2 activation in the liver remains unclear. Moreover, CTRP3 has the ability to regulate glucose metabolism in an insulin-dependent or -independent manner in hepatocytes (Peterson et al., 2010). Certainly, besides the liver, glucose uptake by surrounding tissues is also an important factor affecting blood glucose levels (Cherrington, 1999). *In vitro* experiments show that CTRP3 doesn’t affect glucose uptake and AKT pathway in 3T3-L1 adipocytes and muscle cells (L6 myotubes) (Peterson et al., 2010). The metabolic role of CTRP3 in peripheral tissues needs further exploration.
2. CTRP3 attenuates insulin resistance by regulating lipid metabolism in the liver and AT. In the liver, CTRP3 inhibits the synthesis of triacylglycerols (TAGs), thereby reducing liver lipid content and improving hepatic insulin resistance induced by a high-fat diet (Peterson et al., 2013). In the case of chronic overexpression of transgenic CTRP3 or acute administration of rCTRP3, CTRP3 will reduce the expression of TG synthesis genes (GPAT, AGPAT, and DGAT) in the liver (Peterson et al., 2013). Consistently, CTRP3 effectively prevents both non-alcoholic fatty liver and alcoholic fatty liver (Peterson et al., 2013; Trogen et al., 2018). Consistent with this, under HFD conditions, CTRP3 knockout mice have higher liver TG levels than wild-type mice (Wolf et al., 2016). Interestingly, both transgenic overexpression and genetic deletion of CTRP3 have no significant change in lipid metabolism in low fat-fed mice (Peterson et al., 2013; Wolf et al., 2016), suggesting that CTRP3 may specifically regulate lipid disorders. Additionally, *in vitro* data show that pre-treatment with CTRP3 increases total oxygen consumption in H-4-II-E hepatoma cells under the condition of an excess of free fatty acids. *In vivo* data showed that the respiratory exchange rate of transgenic mice overexpressing CTRP3 was reduced compared with the control group. These data imply that CTRP3 enhances the hepatic fat oxidation, which plays a role in liver lipid metabolism, but the underlying mechanism is unclear. Moreover, we know that the expansion of AT causes insulin resistance (Ota, 2013). CTRP3 reduces adipogenesis in adipocytes by suppressing the expression of adipogenesis-related genes (Kopp et al., 2010; Nishimoto et al., 2017).
3. CTRP3 enhances insulin sensitivity by inhibiting inflammation or regulating insulin signaling transduction. Previous studies have demonstrated that chronic low-grade inflammation plays a critical role in obesity-related IR (Xu et al., 2003; Apovian et al., 2008). Indeed, *in vitro* and *in vivo* data have clarified that CTRP3 decreases the levels of IL-6, MCP-1, TNF, MIF, CCL3/MIP1, and CCL4 in either adipocytes or AT-infiltrated inflammatory cells through inhibiting the binding of LPS to TLR4. Conversely, CTRP-3 knockdown can reduce the release of anti-inflammatory factors and cause adipocytes to dedifferentiate, thereby having a stronger pro-inflammatory ability and lower insulin sensitivity (Kopp et al., 2010). In the 3T3-L1 adipocyte model with IR, rCTRP3 reduces the release of IL-6 and TNF-α by increasing the expression of PKB (ser437) and GLUT-4 and improves glucose consumption, glucose uptake, and insulin sensitivity. It is worth noting that wortmannin (a special inhibitor of PI3K) is sufficient to inactivate these effects, indicating that PI3K / PKB / GLUT4 signaling is involved. However, the overexpression of GLUT4 helps glucose to enter AT too much, thereby increasing the quality of AT (Shepherd et al., 1993).
4. CTRP3 increases the secretion of other adipokines, such as adiponectin, leptin, visfatin, apelin, and resistin, which are beneficial to regulate glucose metabolism or attenuate insulin resistance (Liu et al., 2012; Li et al., 2014a). For example, CTRP3 significantly increases the expression of disulfide bond-A oxidoreductase-like protein (DsbA-L) and adiponectin in 3T3-L1 adipocytes by activating AMPK signaling (Liu et al., 2012); in addition, DsbA-L not only promotes the multimerization of adiponectin, thereby enhancing the biological activity of adiponectin, but also prevents the reduction of adiponectin induced by endoplasmic reticulum stress, thereby improving insulin sensitivity (Pajvani et al., 2003; Liu et al., 2008, 2012; Liu and Liu, 2014).
5. recently, the level of CTRP3 in the circulation is related to β cell function. A study showed that fasting CTRP3 levels are positively associated with HOMA-β (an index for evaluating the function of individual islet β cells) and negatively related to HOMA-IR (an index for evaluating individual’s insulin resistance level), fasting plasma glucose (FPG), 1-h PG, 2-h PG, and fasting plasma C-peptide in the gestational diabetes mellitus patients (the specific cases are unknown) (Li et al., 2017). However, in another study, serum CTRP3 levels in obese children are negatively and significantly correlated with HOMA-IR, HOMA-β, BMI, TG, systolic blood pressure, fasting insulin, and glucose (Zhang and He, 2019). On the other hand, acute injection of rCTRP3 in rodents has a hypoglycemic effect but does not affect insulin levels (Zhang and He, 2019). In summary, the effect of CTRP3 on β cell function remains to be explored.
6. Lastly, Peterson et al. (2010) several studies have reported the association between CTRP3 and diabetic complications. Specifically, in terms of diabetic retinopathy, CTRP3 attenuates HG-induced oxidative stress and apoptosis in ARPE-19 cells, a retinal pigment epithelial cell line, involving the activation of the Nrf2/HO-1 pathway. In addition, CTRP3 reduces the expression of high glucose and high lipid-induced vascular cell adhesion molecule 1 (VCAM-1) in human retinal microvascular endothelial cells involved in the AMPK pathway (Yan et al., 2017). Preclinical investigations revealed that VCAM-1 levels may reflect endothelial cell damage in NIDDM patients with proliferative diabetic retinopathy (Yoshizawa et al., 1998). CTRP3 also enhances the axon growth and protein synthesis rate of spinal muscular atrophy motor neurons through the mTOR pathway, which suggests that CTRP3 may have the potential to improve diabetic neuropathy (Rehorst et al., 2019). Wang F. showed that CTRP3 can reduce the production of inflammatory factors and the number of cell losses induced by high glucose in HUVEC by activating the AKT-mTOR pathway, suggesting that CTRP3 may play a role in diabetes-related endothelial dysfunction (Wang et al., 2019). Moreover, CTRP3 can inhibit high glucose-induced cell proliferation and extracellular matrix production by inactivating the JAK2/STAT3 pathway in human mesangial cells (Hu et al., 2019), indicating that CTRP3 may be a therapeutic target for diabetic nephropathy. In diabetic cardiomyopathy (DCM), CTRP3 reduced inflammation, oxidative stress, and cell death in DCM rats, and improved cardiac dysfunction (Ma et al., 2017).

Currently, the information shows that CTRP3 inhibits the synthesis of glycogen and triglycerides in the liver, increases lipid oxidation in the liver, reduces fat synthesis in adipose tissue, and inhibits systemic inflammation, thereby participating in the regulation of blood sugar homeostasis, reducing insulin resistance and prevention the development of diabetes complications.

## CTRP3 in Cardiovascular Disorders

Cardiovascular disease is the most common cause of death worldwide, which accounts for roughly 40% of the total death toll (Dagenais et al., 2020). Novel biomarkers are needed to be identified, which can also be used as a new treatment for combating CVD. Recently, CTRP3 has been reported to negatively correlate with lots of cardiometabolic risk factors (Yoo et al., 2013; Choi et al., 2014), such as high blood pressure and obesity (Deng et al., 2015; Wagner et al., 2016). *In vivo*, the level of circulating CTRP3 decreased significantly one day after MI, reached the lowest point, and then slowly recovered. On the first day after MI, the level of CTRP3 mRNA in adipocytes decreased significantly, and then gradually recovered (Akiyama et al., 2007). Zhu H. et al. (Zhu et al., 2020) demonstrated that CTRP3 may promote the conversion of monocytes to an anti-inflammatory phenotype post-MI. CTRP3 levels also decrease in patients with stable angina pectoris (Choi et al., 2014), acute coronary syndrome, acute aortic dissection (Jiang et al., 2018), and heart failure either with reduced ejection fraction (Gao et al., 2019) or with preserved ejection fraction (Liu et al., 2018) compared with control subjects. Notably, excessive CTRP3 may be harmful to cardiovascular health since CTRP3 promotes phosphate-induced osteogenic differentiation of vascular smooth muscle cells (Zhou et al., 2014), causing vascular calcification. An increase in vascular calcification accelerates arterial hardening and aging (Xu et al., 2020). Therefore, maintaining CTRP3 at an appropriate level is a key factor in preventing cardiovascular disease. In addition, a study showed that proper exercise can reduce arterial stiffness in middle-aged and elderly people and increase serum CTRP3, adiponectin, and CTRP5 (Hasegawa et al., 2018). Another study showed that CTRP3 may effectively reduce the inflammation caused by oxidized LDL in mouse aortic endothelial cells and improve endothelial dysfunction by activating PI3K/Akt/eNOS signaling, which indicates a promising atheroma Sclerotherapy strategy (Chen et al., 2019).

Available evidence shows that CTRP3 mainly protects the cardiovascular system in the following two ways, namely promoting angiogenesis and improving inappropriate cardiovascular remodeling after MI. Angiogenesis relies on an extensive signal transduction network among and within endothelial cells (ECs), correlative parietal cells (such as VSMCs), and other cell types (such as immune cells). According to the report, CTRP3 promotes cell proliferation and migration via activating ERK1/2 in mouse endothelial cells (MSS31 cells) (Akiyama et al., 2007). Peterson JM et al. found that CTRP3 induces angiogenesis *in vivo* via VEGF-Akt-HIF1 signaling rather than AMPK signaling, as evidence by an increased number of CD31^+^ capillary vessels and α-SMA^+^ arterial density, but not *in vitro* (HUVEC) (Yi et al., 2012). Interestingly, the conditioned medium of primary cardiomyocytes treated with CTRP3 can induce the formation of HUVEC tubes (the formation of capillary-like structures), which suggests that CTRP3 may induce cardiomyocytes to secrete some paracrine factors (Yi et al., 2012). However, we can’t completely rule out the direct effect of CTRP3 on HUVEC, as Yi et al. (2012) increased CTRP3 levels using bacteria in their *in vitro* experiments and using adenovirus vectors in their *in vivo* experiments. This divergence suggests that the effects of CTRP3 on vascular endothelium may involve post-translational modification. As for another important cell in angiogenesis -VSMCs, TGF-β1 induces the expression of CTRP3 in VSMCs (p53LMAC01). rCTRP3 promotes VSMCs proliferation in a dose-dependent manner by activating ERK1/2 and MAPK signaling. By contrast, rCTRP3 doesn’t alter VSMCs migration (Maeda and Wakisaka, 2010). On the other hand, Peterson JM et al. found that CTRP3 improved the left ventricular (LV) systolic and diastolic function of MI animals, improved survival rate, and prevented LV heart remodeling. It is worth noting that CTRP3 treatment significantly increased the ratio of cardiomyocytes to fibrotic cells in ischemic areas, reduced the size of cardiomyocytes in non-ischemic areas, and significantly reduced the interstitial fibrosis by blocking TGF β signaling (Yi et al., 2012). Additionally, the mesenchymal stromal cells (MSCs) transplantation provides a promising choice in MI remedy. Recently, Zhang Z. et al. revealed that MSCs express and secrete CTRP3 which increases the survival and retention of MSCs in the ischemia zone post-MI. MSCs with the overexpression of CTRP3 potentiates the protective effects of MSCs on cardiac post-MI via improving pathologic remodeling, and promoting angiogenesis, possibly involving the activation of ERK1/2 and the up-regulation of MMP9 and MT1/2/SOD2 (Zhang Z. et al., 2019). CTRP3 reduces cardiac fibrosis via suppressing myofibroblast differentiation and extracellular matrix production. Specifically, CTRP3 reduces adventitial fibroblast phenotypic conversion (Lin et al., 2014), cell proliferation and migration, and expressions of profibrotic molecules (such as connective tissue growth factor and collagen I) induced by TGF-β1 in cardiac fibroblasts (Wu et al., 2015). In addition, CTRP3 counteracts the phosphorylation of smad3 induced by TGF-β1 to prevent nuclear translocation of p-smad3 and further interaction with p300 (Wu et al., 2015). Although AMPK signaling is not necessary for angiogenesis in the previous report (Yi et al., 2012), it plays a key role in the anti-fibrotic effect of CTRP3 after MI by activating smad3 (Wu et al., 2015).

In addition to the substantial contribution in MI, CTRP3 also directly influences the cardiomyocytes or ECs in different ways in other types of CVD. In both animal models and cultured cells, CTRP3 has a protective effect on stroke (intracerebral hemorrhage) (Wang et al., 2016; Yang et al., 2017). CTRP3 stimulates mitochondrial biogenesis in cardiomyocytes via AMPK/PGC-1α signaling (Zhang et al., 2017b) and significantly induces vasorelaxation (Zheng et al., 2011). CTRP3 increases cardiomyocyte contractility by enhancing calcium sensitivity (Zhang et al., 2017a). Exosomal miRNAs that mediate cell-to-cell communication in many fields (Cui et al., 2012; Shan et al., 2019; Wu et al., 2019; Xu et al., 2020), contribute to CTRP3 gene expression, thereby regulating the proliferation and apoptosis of cardiomyocytes induced by HG via activating JNK signaling (Song et al., 2020). However, the implications of CTRP3 in cardiac hypertrophy have yet to be explored, as the reported results are contradictory in the literature. Some studies found that CTRP3 attenuates diabetes-related cardiomyopathy by activating AMPKα (Ma et al., 2017) and attenuates pressure overload-induced cardiac hypertrophy in the animal model (Zhang B. et al., 2019). Briefly, CTRP3 deficiency impairs the cardiac contractile dysfunction, exacerbates cardiomyocytes enlargement and myocardial fibrosis, and reprograms the expression of pathological genes after transverse aortic constriction (TAC) surgery compared with WT mice. In contrast, CTRP3 overexpression partly rescues these effects. In addition, CTRP3 improves left ventricular dysfunction and inappropriate cardiac remodeling via suppressing the p38/CREB signaling and attenuating p38 MAPK-induced endoplasmic reticulum stress (Zhang B. et al., 2019). Paradoxically, another experiment indicates that CTRP3 aggravates cardiac hypertrophy induced by pressure overload due to aortic banding (AB) (Ma et al., 2019). Briefly, CTRP3 expression of cardiomyocytes induced by reactive oxygen species is up-regulated in murine cardiac hypertrophy model and heart failure patients. The cardiac dysfunction and hypertrophy are aggravated in CTRP3-overexpressing mice using adeno-associated virus vector, whereas CTRP3 deficiency in the heart alleviates hypertrophic phenotype. *In vitro*, CTRP3 directly induces hypertrophy in cardiomyocytes and the underlying mechanism is that CTRP3 exacerbates cardiac hypertrophy via the TGF β-activated kinase 1 (TAK1)-c-Jun N-terminal kinase (JNK) axis which is activated by PKA (Ma et al., 2019). Regardless, more research is needed to explain this conflict. Recently, Wei WY and colleagues reported that CTRP3 prevents sepsis-induced cardiomyopathy in mice by activating AMPKα signaling (Wei et al., 2018) and ameliorates doxorubicin-induced cardiomyopathy via activating Sirt1 (Yuan et al., 2018).

Considering the above evidence, CTRP3 not only promotes angiogenesis and improves inappropriate cardiovascular remodeling after MI, but also ameliorates the function of damaged endothelial cells and cardiomyocytes. However, the role of CTRP3 in cardiovascular disease needs further studies to support it.

## Conclusion

**FIGURE 2:**
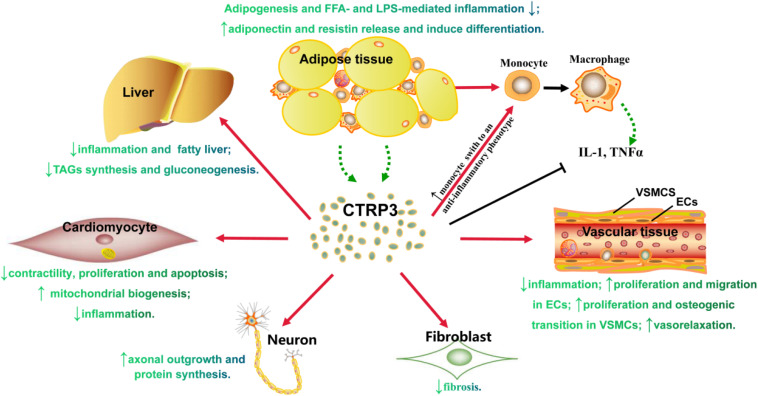
The role of CTRP3 in either tissue or cells. Triacylglycerol (TAGs).

Adipokines secreted by AT contribute to various biological processes. CTRP3 is a new type of adipokine that can affect obesity-related cardiovascular diseases (Figure 2), which provides extensive academic research and is considered a promising therapeutic strategy. However, the following areas require further investigation: (Rosen and Spiegelman, 2006) continuous monitor of the change of CTRP3 levels in normal people, sub-clinical stage, prodromal period, clinical stage, and treatment period, and notably, excessive CTRP3 levels possibly promoting vascular calcification; (Scherer, 2006) the influences of CTRP3 on non-diabetes-related cardiomyopathy using a different method to establish study model or diabetes-related cardiomyopathy; (Akoumianakis and Antoniades, 2017) the effects of CTRP3 on pancreatic β cell, adipocyte differentiation or dedifferentiation, and muscle; (Kishore et al., 2004) the potential adverse effects: CTRP3 stimulates osteosarcoma tumor growth (Akiyama et al., 2009), and promotes the transition of prostate cells into cancer cells (Hou et al., 2015). Thus, all research results should be comprehensively evaluated before developing CTRP3 as an effective therapeutic target.

## Author Contributions

L-QY conceived and revised the manuscript. BG, TZ, FX, XL, S-KS, FW, J-YZ, FL, YW, M-HZ, and Q-SX analyzed the data. BG wrote the manuscript. TZ, Q-SX, and UE revised the manuscript. All authors contributed to the article and approved the submitted version.

## Conflict of Interest

The authors declare that the research was conducted in the absence of any commercial or financial relationships that could be construed as a potential conflict of interest.

## Abbreviations

AB: aortic banding
AT: adipose tissue
BMI: body mass index
CTRP3: C1q tumor necrosis factor-related protein 3
CTRPs: C1q tumor necrosis factor-related proteins
CVD: cardiovascular disorders
C/EBPα: CCAAT/enhancer binding protein alpha
CCL: CC-chemokine ligand
JNK: c-JunN-terminal kinase
CORS26: collagenous repeat-containing sequence of 26-kDa protein
CRP: C-reactive protein
DM: diabetes mellitus
DCM: diabetic cardiomyopathy
DIO: diet-induced obesity
DsbA-L: disulfide bond-A oxidoreductase-like protein
FBG: fasting blood glucose
FPG: fasting plasma glucose
FABP4: fatty acid binding protein 4
GLP-1: glucagon-likepeptide-1
HFD: high-fat diet
HUVEC: human umbilical vein endothelial cells
IR: insulin resistance
JAK: just another kinase or janus kinase
LV: left ventricle
LPS: Lipopolysaccharide
LDL: low density lipoprotein
LAMP-1: lysosomal-associated membrane protein 1
LIMP II: lysosome membrane protein 2
(MIP)-1: macrophage inflammatory protein
MIF: macrophage migration inhibitory factor
mTOR: mammalian target of rapamycin
MSCs: Mesenchymal stromal cells
MCP-1: monocyte chemoattractant protein-1
PPARγ: peroxisome proliferator-activated receptor gamma
PKA: protein kinase A
rCTRP3: recombinant CTRP3 protein
STAT3: signal transducers and activators of transcription 3
TXA2: thromboxane A2
TLR4: Toll-like receptor 4
TGF-β1: transforming growth factor β 1
TAK1: transforming growth factor β -activated kinase 1
TAC: transverse aortic constriction
TG: triglyceride
TNF: tumor necrosis factor
T2DM: type 2 diabetes mellitus
VCAM-1: vascular cell adhesion molecule-1.
